# Supplementary material for: Identification of a Hypomorphic FANCG Variant in Bernese Mountain Dogs
Source: Genes (Basel). 2022 Sep 21;13(10):1693. doi: 10.3390/genes13101693 (PMC9601343; doi:10.3390/genes13101693)

**Figure S1. Whole exome sequencing of a Bernese Mountain Dog identifies a variant in the *FANCG* gene at the CFA11:51655193 position when aligned to the CanFam3.1 canine reference genome.** The figure depicts the alignment generated in Integrative Genomics Viewer (IGV) (https://software.broadinstitute.org/software/igv/). At the CFA11:51655193 position, 9/10 reads have a C instead of the reference T, resulting in a Q>R amino acid substitution. Note that the gene is encoded on the reverse strand.

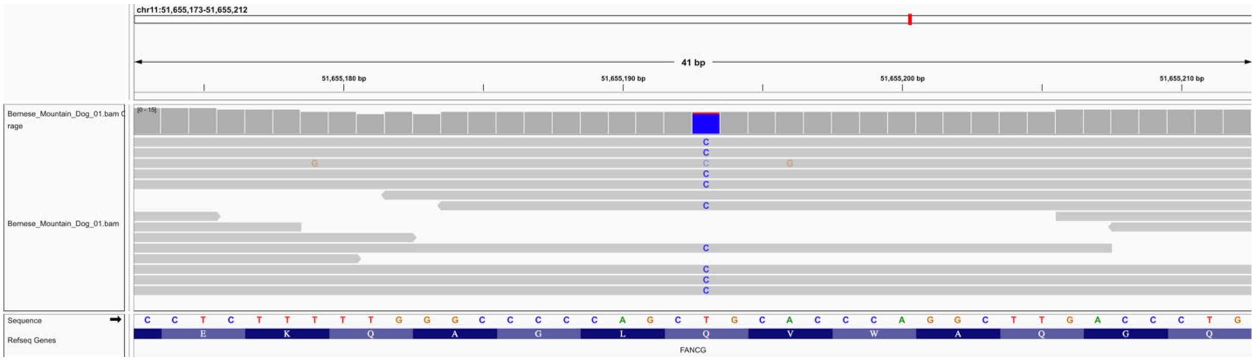

Supplement: Supplementary file 1 [file genes-13-01693-s001.zip › Figure S1.pdf]
