# Supplementary material for: Identification of a Hypomorphic FANCG Variant in Bernese Mountain Dogs
Source: Genes (Basel). 2022 Sep 21;13(10):1693. doi: 10.3390/genes13101693 (PMC9601343; doi:10.3390/genes13101693)
Supplement: Supplementary file 1 [file genes-13-01693-s001.zip › Figure S2.pdf]

Figure S2. FANCG protein sequence alignment comparing human and dog. The human sequence (Human-201, Ensembl transcript ENST00000378643.8) was obtained from the GRCh38 genome assembly. The dog sequences (Dog-201 and Dog-202, Ensembl transcripts ENSCAFT00000003158.5 and ENSCAFT00000102034.1) were obtained from the CanFam3.1 genome assembly. The fourth row indicates similarity or difference between the protein sequences (an asterisk indicates positions of conservation, a colon indicates conservation between groups of strongly similar properties, a period indicates conservation between groups of weakly similar properties). The FANCG variant ^{465}R is bolded in **red** in all transcripts. The seven referenced tetratricopeptide repeats are highlighted, alternating in turquoise and yellow in the human sequence [40]. All protein sequences were generated from Ensembl transcripts (http://www.ensembl.org). Sequence alignment was performed using Clustal Omega sequence alignment software (http://www.clustal.org/omega).

```
Human-201      ------------------------------------------------------------ 0
Dog-201        MPCAGPPPAEGQSWADLEAWLRAEGWDTVSGTEIDCSRFRPARGTGKPNREKYNLPTQTH 60
Dog-202        ------------------------------------------------------------ 0


Human-201      ------------------------------------------------------------ 0
Dog-201        PHPLVRRRLPFRMESPHVSSSSFPRGLTLEDDQPQQRVLSAGPTRGEMEEAGVGAGTESP 120
Dog-202        ------------MESPHVSSSSFPRGLTLEDDQPQQRVLSAGPTRGEMEEAGV------- 41


Human-201      ------------------------------------------------------------ 0
Dog-201        RESISPEKADSPLRGPSGEQKSRGRVRKRRDLSPLGWEPRRDRPYPSADPSRCPGPASAT 180
Dog-202        ------------------------------------------------------------ 41


Human-201      ----MSRQTTSVGSSCLDLWREKNDRLVRQAKVAQNSGLTLRRQQLAQDALEGLRGLLHS 56
Dog-201        ISHQTPLSSSGPHTSCLDLWREKNDQLVRQAKVAQDSRLYVRRQQLAQDALEGFRGLLHS 240
Dog-202        ---------------------------------AQDSRLYVRRQQLAQDALEGFRGLLHS 68
                                                **:* * :************:******

Human-201      LQGLPAAVPVLPLELTVTCNFIILRASLAQGFTEDQAQDIQRSLERVLETQEQQGPRLEQ 116
Dog-201        LQGLPAAVPVLPLELTVTCNFITLRATLAQGFTEDQAQDIQKGLERVLETQEQLGPRLEC 300
Dog-202        LQGLPAAVPVLPLELTVTCNFITLRATLAQGFTEDQAQDIQKGLERVLETQEQLGPRLEC 128
               ********************** ***:**************:.********** *****

Human-201      GLRELWDSVLRASCLLPELLSALHRLVGLQAALWLSADRLGDLALLLETLNGSQSGASKD 176
Dog-201        GLRGLWDSVFHYSSLLLELLPVLHHLAGLQAALWLSTDHLGDLTLLLQTLNGKQSEASEN 360
Dog-202        GLRGLWDSVFHYSSLLLELLPVLHHLAGLQAALWLSTDHLGDLTLLLQTLNGKQSEASEN 188
               *** *****:: *.** *** .**:*.*********:*:****:***:****.** **::

Human-201      LLLLLKTWSPPAEELDAPLTLQDAQGLKDVLLTAFAYRQGLQELITGNPDKALSSLHEAA 236
Dog-201        LLLLLKTWSPPPKESDAPLTLQDARGLRDVLLTASAYRQGLQELITGSLPRALSSLQEAA 420
Dog-202        LLLLLKTWSPPPKESDAPLTLQDARGLRDVLLTASAYRQGLQELITGSLPRALSSLQEAA 248
               *********** :* *********:**:****** ************.  :*****:***

Human-201      SGLCPRPVLVQVYTALGSCHRKMGNPQRALLYLVAALKEGSAWGPPLLEASRLYQQLGDT 296
Dog-201        SGLCSRPVLVQVYTALGTCLHKMGNPQRALLYLVAALKEGSTWGLPLLEASRLYRQLGNT 480
Dog-202        SGLCSRPVLVQVYTALGTCLHKMGNPQRALLYLVAALKEGSTWGLPLLEASRLYRQLGNT 308
               **** ************:* :********************:** *********:***:*

Human-201      TAELESLELLVEALNVPCSSKAPQFLIEVELLLPPPDLASPLHCGTQSQTKHILASRCLQ 356
Dog-201        AAELESLELLVEALNVTHSSEAPQLLIEVELLLPQPNPGSPLHCGTQSQAKYLLASRCLQ 540
Dog-202        AAELESLELLVEALNVTHSSEAPQLLIEVELLLPQPNPGSPLHCGTQSQAKYLLASRCLQ 368
               :***************  **:***:********* *: .**********:*::*******
```

```
Human-201       TGRAGDAAEHYLDLLALLLDSSEPRFSPPPSPPGPCMPEVFLEAAVALIQAGRAQDALTL  416
Dog-201         VGRAEDAAEHYLDLLALLLSDSEPKFSPPPTHPGPCMPEVFLEAAAALIQAGRAQDALTV  600
Dog-202         VGRAEDAAEHYLDLLALLLSDSEPKFSPPPTHPGPCMPEVFLEAAAALIQAGRAQDALTV  428
                .*** **************..***:*****: *************.*************:

Human-201       CEELLSRTSSLLPKMSRLWEDARKGTKELPYCPLWVSATHLLQGQAWVQLGAQKVAISEF  476
Dog-201         CEELLSRTSSLLPKMPQLWEDDKKGTKKSPHCPSWVSATYLLQGQAWVQLGAQKEAISEF  660
Dog-202         CEELLSRTSSLLPKMPQLWEDDKKGTKKSPHCPSWVSATYLLQGQAWVQLGAQKEAISEF  488
                *************** :**** :****: *:** *****:************** *****
                                                              Q>R

Human-201       SRCLELLFRATPEEKEQGAAFNCEQGCKSDAALQQLRAAALISRGLEWVASGQDTKALQD  536
Dog-201         SRCLELLFRTTPKDKEQGPASNGEQGCMSDVALPQLRAAALISRGLQWVASGQDTRALQD  720
Dog-202         SRCLELLFRTTPKDKEQGPASNGEQGCMSDVALPQLRAAALISRGLQWVASGQDTRALQD  548
                *********:**::**** * * **** **.** ************:********:****

Human-201       FLLSVQMCPGNRDTYFHLLQTLKRLDRRDEATALWWRLEAQTKGSHEDALWSLPLYLESY  596
Dog-201         FLLGVQMCPGNQDASFHLLQTLRKMDRRDEASALWWRLEAQTKLPQENAAWSLPLYLETC  780
Dog-202         FLLGVQMCPGNQDASFHLLQTLRKMDRRDEASALWWRLEAQTKLPQENAAWSLPLYLETC  608
                ***.*******:*: *******:::******:***********  :*:* ********:

Human-201       LSWIRPSDRDAFLEEFRTSLPKSCDL  622
Dog-201         LGWICYPDRETLLEEFRTSLPEPCDL  806
Dog-202         LGWICYPDRETLLEEFRTSLPEPCDL  634
                *.**   **:::*********: ***
```
