# Supplementary material for: Identification of a Hypomorphic FANCG Variant in Bernese Mountain Dogs
Source: Genes (Basel). 2022 Sep 21;13(10):1693. doi: 10.3390/genes13101693 (PMC9601343; doi:10.3390/genes13101693)
Supplement: Supplementary file 1 [file genes-13-01693-s001.zip › Figure S3.pdf]

Figure S3. FANCG 5′ UTR and exon 1 cDNA sequence alignment comparing human and dog. The human sequence (Human-201, Ensembl transcript ENST00000378643.8) was obtained from the GRCh38 genome assembly. The dog sequence (Dog-201, Ensembl transcript ENSCAFT00000003158.5) was obtained from the CanFam3.1 genome assembly. The predicted start codons are highlighted (Human-201 in turquoise, Dog-201 in yellow). The third row indicates positions of nucleotide conservation (asterisk) (64% in the 5′ UTR of the human as compared to the dog coding region). All DNA sequences were generated from Ensembl transcripts (http://www.ensembl.org). Sequence alignment was performed using Clustal Omega sequence alignment software (http://www.clustal.org/omega).

```
Human-201       ------------------------------------------------------------     0
Dog-201         ATGCCCTGCGCGGGCCCGCCCCCTGCGGAGGGGCAAAGCTGGGCCGACCTGGAAGCGTGG     60

Human-201       ------------------------------------------CCTTTCTCGAGGCTGTGG     18
Dog-201         CTGCGAGCGGAGGGCTGGACACGGTTTCCGGTACCGAGATCGATTGCTCAAGGTTTCGG     120
                                                            ** *** *** *  **

Human-201       CCTCCGCGAGAGCCGAGCGGGCCGCACCGCCGGCCGTGCGACTGCCCCAGTCAGACACGA     78
Dog-201         CCTGCGCGAGGGACTGGCAAACCGAACCGTGAGAAGTACAACCTCCCCACTCAGACGCAC     180
                *** ****** * *  **   *** ****   *  ** * **  ***** ****** *

Human-201       CCCCGGCTTCTAGCCCGCCTA-------AGCCTGTTTGGGGTTGCTGACTCGTTTCCTCC     131
Dog-201         CCCCATCCTCTGGTCCGCCGTCGGCTCCCTTTTCGGATGGAGAGCCCACACGTTTCTTCC     240
                ****  * *** * *****             *     **   **  ** ****** ***

Human-201       CCGAGTTTCCCGCGGGAACTAACTCTTCAAGAGGACCAACCGCAGCCCAGAGCTTCGCAG     191
Dog-201         TCGAGTTTCCCGCGAGGACTAACCCTTGAGGACGACCAACCGCAGCAGAGAGTTTTGTCC     300
                 ************* * ****** *** * ** *************  **** ** *

Human-201       ACCCGGCCAACCAGAGGCGAGGTTGAGAGCCCGGCGGGCCGCGGGGAGAGAGCGTCCCAT     251
Dog-201         GCGGGGCCAACCAGAGGCGAGATGGAGGAGGCGGGGGTGGGAGCCGGCACAGAGTCCCCG     360
                 *  ***************** * ***    *** **   * *  *  * ** *****

Human-201       CT-----------GTCCTGGAA-AGCCTGGGCGGGTGGATTGGGACCCC-GAGAGAAGCA     298
Dog-201         AGAGAGTCCATTTCTCCCGAAAAGGCCGATTCGCCTCTGCGGGGGCCCTCAGGGGAGCAA     420
                              *** * **  ***    **  *     *** ***    * **   *

Human-201       GGGGAGCTCGGCGGGGTGCAGAAGTGCCCAGGCCCCTCCCCGCTGGGGTTGGAGCTTGG     358
Dog-201         AAGAGTCGCGGGAGGGTGCGGAAGCGCAGGGACCTTTCCCCGCTCGGGTGGGAACCGCGG     480
                  *   * ***  ****** **** **   * **  ******** **** ** * *  **

Human-201       GCAGGCCAGCTTCACCCTTCCTAAGTCCGCTTCTGGTCTCCGGGCCCAGCCTCGGCCACC     418
Dog-201         CGGGACCGGCCTTACCCTTCAGCAGACCCTTCCAGGTGTCCGGGTCCAGCCTCGGCCACC     540
                   * ** ** * *******   ** **  * * *** ****** ***************

Human-201       ATGTCCCGCCAGACCACCT------------CTGTGGGCTCCAGCTGCCTGGACCTGTGG     466
Dog-201         ATATCGCATCAGACCCCTCTGAGCTCCTCAGGGCCACACACCAGCTGCCTAGACTTGTGG     600
                ** ** *  ****** *                     * ********** *** *****

Human-201       AGGGAAAAGAATGACCGGCTCGTTCGACAGGCCAAG   502
Dog-201         AGGGAAAAGAATGACCAGCTAGTTCGACAGGCCAAG   636
                **************** *** ***************
```
