# Supplementary material for: Identification of a Hypomorphic FANCG Variant in Bernese Mountain Dogs
Source: Genes (Basel). 2022 Sep 21;13(10):1693. doi: 10.3390/genes13101693 (PMC9601343; doi:10.3390/genes13101693)

Figure S4. Cumulative age distribution at diagnosis for histiocytic sarcoma affected Bernese mountain dogs. (**A**) Data are from 121 HS affected dogs from our repository at Michigan State University included in this study. (**B**) Data are from 1818 HS affected dogs from the Berner Garde database reflecting cases reported to the database with a veterinarian confirmed diagnoses as of January 2020.

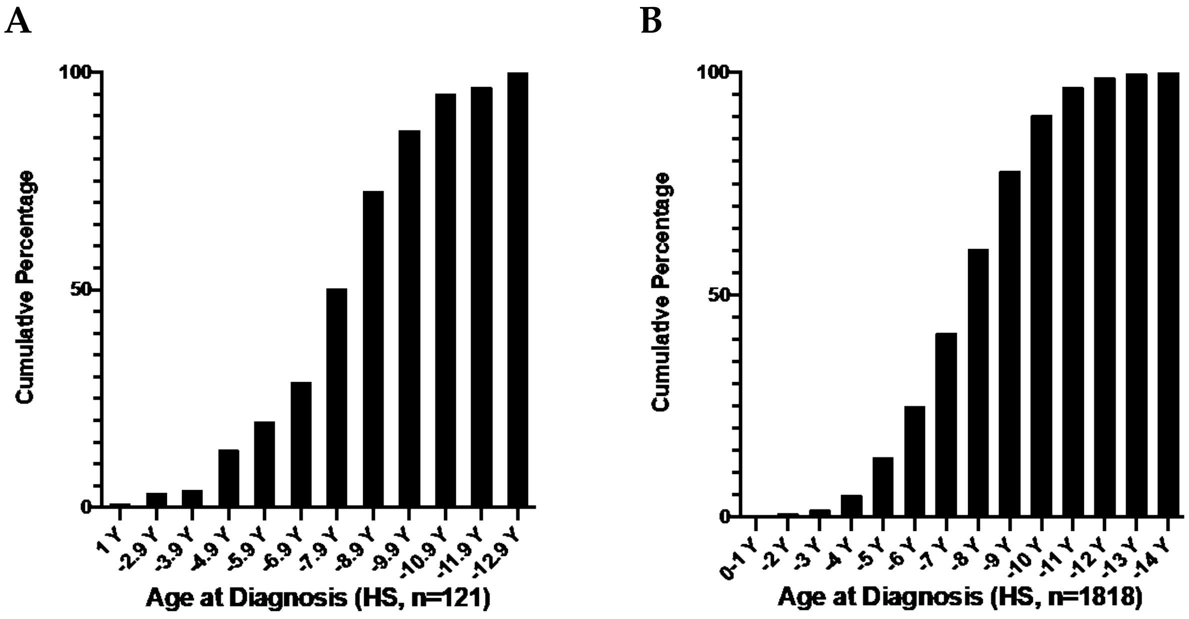

Supplement: Supplementary file 1 [file genes-13-01693-s001.zip › Figure S4.pdf]
