# Supplementary material for: Identification of a Hypomorphic FANCG Variant in Bernese Mountain Dogs
Source: Genes (Basel). 2022 Sep 21;13(10):1693. doi: 10.3390/genes13101693 (PMC9601343; doi:10.3390/genes13101693)

Figure S5. Distribution of genotype and (**A**) height at withers and (**B**) weight in healthy male BMDs, over 1 years of age. Dogs that are homozygous for the variant (n=2, ages 6.8 and 6.9 yrs), heterozygous, (n=8, ages 1.3-8.6 yrs), and homozygous for the reference (n=3, ages 3.3-8.6 yrs) *FANCG* alleles are indicated by the solid black bars, the checkered bars and the solid white bars, respectively.

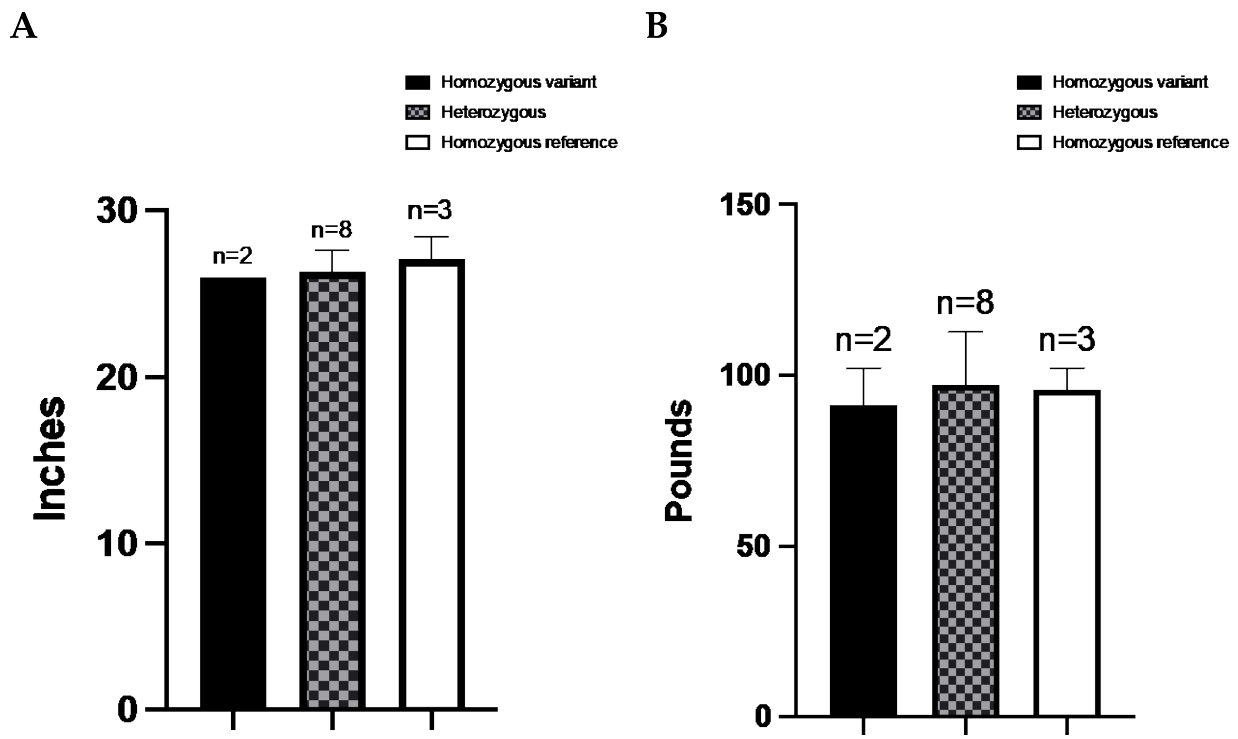

Supplement: Supplementary file 1 [file genes-13-01693-s001.zip › Figure S5.pdf]
