# Supplementary material for: Identification of a Hypomorphic FANCG Variant in Bernese Mountain Dogs
Source: Genes (Basel). 2022 Sep 21;13(10):1693. doi: 10.3390/genes13101693 (PMC9601343; doi:10.3390/genes13101693)

Figure S6. Three-dimensional structure of a subsection of the human FANCG protein. The Q465 amino acid is highlighted in green on the 3D structure. The 3D image was generated using AlphaFold, accessed via the UniProtKB database (https://www.uniprot.org/).

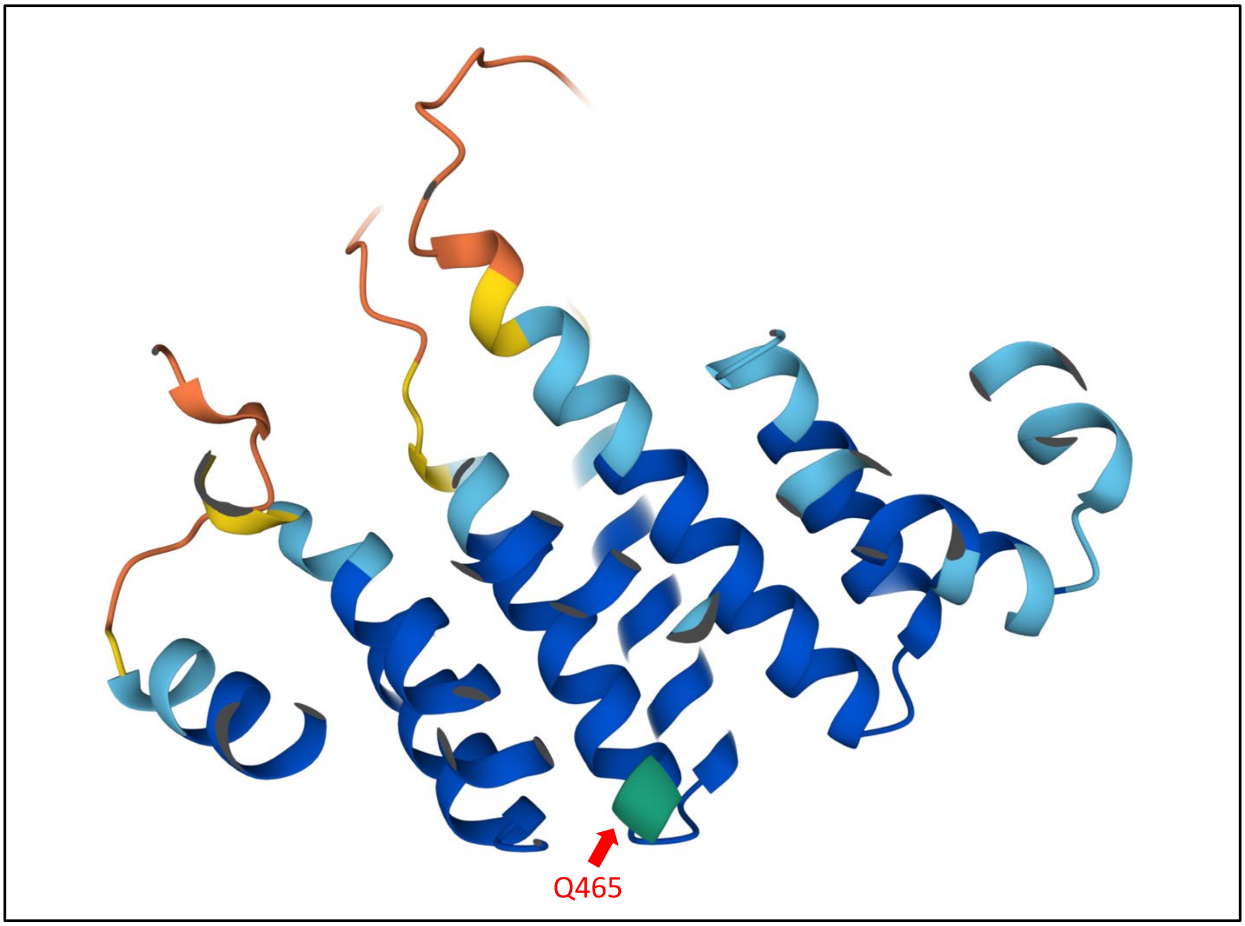

Supplement: Supplementary file 1 [file genes-13-01693-s001.zip › Figure S6.pdf]
