# Supplementary material for: Identification of a Hypomorphic FANCG Variant in Bernese Mountain Dogs
Source: Genes (Basel). 2022 Sep 21;13(10):1693. doi: 10.3390/genes13101693 (PMC9601343; doi:10.3390/genes13101693)

Figure S7. Ablation of DNA-PKcs in FANCG-/- 293T cells partially restores cisplatin resistance. (**A**) Immunoblot for DNA-PKcs or XRCC4 expression in wild-type 293T cells, *FANCG-/-* 293T cells, or *FANCG-/-* 293T cells that were CRISPR targeted to ablate DNA-PKcs expression. DNA-PKcs targeting was first assessed by PCR of targeted region, and DNA-PKcs expression confirmed by immunoblotting. (**B**) Wild type 293T cells, *FANCG-/-*, and *FANCG-/-* cells that were either deficient or haplo-insufficient for DNA-PKcs were tested for cisplatin sensitivity. Cell viability was assessed by MTS assay and is expressed as % survival of untreated controls. Assay is representative of four independent experiments, each performed in triplicate. Error bars represent SEM.

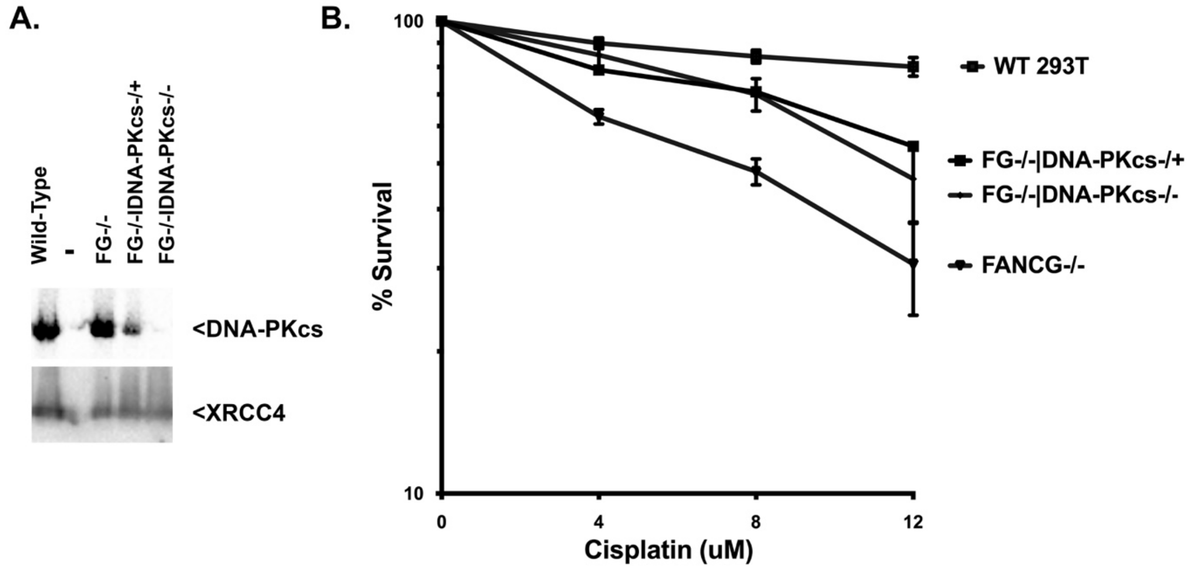

Supplement: Supplementary file 1 [file genes-13-01693-s001.zip › Figure S7.pdf]
