# Supplementary material for: Identification of a Hypomorphic FANCG Variant in Bernese Mountain Dogs
Source: Genes (Basel). 2022 Sep 21;13(10):1693. doi: 10.3390/genes13101693 (PMC9601343; doi:10.3390/genes13101693)
Supplement: Supplementary file 1 [file genes-13-01693-s001.zip › Table S1.pdf]

Table S1. Sequence information for each gRNA and the PCR primers used.

FANCG 465R PAM	GGGCCAGGCCTGGGTTCAAC
FANCG 465R HDR	CATACTGCCCACTCTGGGTCTCTGCCACCCACCTGCTTCAG GGCCAGGCCTGGGTTAGGCTGGGTGCCCAAAAAGTGGCAA TTAGTGAATTTAGCAGGTGAGCCCGGGGTCCTAGAGGGG
FANCG 71P PAM	GCAGGTGACAGTCAGCTCCA
FANCG 71P HDR	CAGTCTGTAAGACCAACATTGTTGCTCCAGGGCTCCCTGCA GCTGTTCCTGTTCTTCCCTTGGAGCCGACGGTCACCTGCAA CTTCATTATCCTGAGGGCAAGCTTGGCCCAGGGTTTCA

Sequences of oligonucleotides use in PCR:

5′ *FANCG* Q465	CCCAAGATGTCCCGGCTGTGGG
3′ *FANCG* Q465	TCTAGGACCCCGGGCTCACCTG
5′ *FANCG* L71	GGGGTGATCTCTGAGTTGGG
3′ *FANCG* L71	AGATGGCAGGGGAATCAGGG
