# Supplementary material for: Identification of a Hypomorphic FANCG Variant in Bernese Mountain Dogs
Source: Genes (Basel). 2022 Sep 21;13(10):1693. doi: 10.3390/genes13101693 (PMC9601343; doi:10.3390/genes13101693)
Supplement: Supplementary file 1 [file genes-13-01693-s001.zip › Table S2.pdf]

Table S2. Complete blood count (CBC) summary and FANCG genotype for a subset of 15 unaffected Bernese mountain dogs. Eight dogs were heterozygous for the variant allele, 4 dogs were homozygous for the variant allele, and 3 dogs were homozygous for the reference allele (0 = reference allele, 1 = variant allele).

Sample ID	Sex	Age (years, months)	Height (inches)	Weight (pounds)	CBC Status	FANCG Allele	MTAP Allele
Dog 01	Female	01 yrs. 01 mos.	24.5	70	Normal	1,1	0,0
Dog 02	Female	10 yrs. 04 mos.	25	93	MPV high	1,1	0,0
Dog 03	Male	06 yrs. 08 mos.	26	83.6	PDW low	1,1	0,0
Dog 04	Male	03 yrs. 11 mos.	25.25	89	Normal	0,1	0,0
Dog 05	Male	03 yrs. 10 mos.	26	99	Monocytes high	0,1	0,0
Dog 06	Female	00 yrs. 09 mos.	26.25	84	Normal	0,0	0,1
Dog 07	Male	01 yrs. 04 mos.	25.25	92	Normal	0,1	0,0
Dog 08	Male	05 yrs. 05 mos.	27.75	102	Reticulocytes minimally high	0,0	0,0
Dog 09	Male	03 yrs. 04 mos.	28	96.2	Normal	0,0	0,1
Dog 10	Male	01 yrs. 10 mos.	28	111.1	MPV high	0,1	0,0
Dog 11	Female	02 yrs. 09 mos.	25.5	97.4	Normal	1,1	0,0
Dog 12	Male	04 yrs. 06 mos.	27.5	115	Normal	0,1	0,0
Dog 13	Female	07 yrs. 00 mos.	27	124.4	MPV high	0,1	0,0
Dog 14	Male	07 yrs. 00 mos.	27.5	89.8	Normal	0,1	0,1
Dog 15	Male	08 yrs. 07 mos.	24.5	76.1	Normal	0,1	0,0

MPV = mean platelet volume
PDW = platelet distribution width
